# A Grippe Characteristic

**Published:** 1894-07

**Authors:** Edward Cranch

**Affiliations:** Erie, Pa.


### A GRIPPE CHARACTERISTIC.

EDWARD CRANCH, M. D., ERIE, PA.

Having been, like all the doctors of my time and generation, ignorant of the behavior of that epidemic neurosis called the grippe or Russian influenza, I have studied it closely, so as to know it from all other manifestations of disease; and I have found its most constant sign, in Erie at least, to be a crimson border to the tongue. It is present in the onset, and throughout the case, persisting until all febrile action has departed. It is not peculiar by reason of its location or extent, so much as by the special tint of color in it. It is emphatically a "crimson lake," and any one with an eye for color will in a short time recognize it, and distinguish it from the scarlet tint of simple fever, or the cyanotic hue of malignant diphtheria, or the pallor of anæmia, in short, it is a color by itself, from whose presence or absence it is safe to say that your case is or is not the grippe. There is always more or less fever present, and generally aching, with debility or prostration, anosmia, agensia, anorexia, in some cases cough; but the first glance at the tongue is enough to decide me in calling the case grippe, and I do not recall a case where the crimson lake border of the tongue was absent, though more or less intense in different subjects.

The explanation of the symptom is in the inflamed state of the capillary system, which was pointed out by Choate, who called the grippe a "capillaritis universalis." I should go back of this, and call it an epidemic neurosis, which causes fever and capillaritis by a partial paralysis of the vaso-motor function; hence the deeper layers of the tongue being affected, the peculiar tint occurs, though why it is limited, as it is, to the bordor of the tongue, is probably explainable by the thinner coat of mucous membrane there, as well as by the local distribution of nerves.

